# How Will AI Shape the Future of Pandemic Response? Early Clues From Data Analytics

**DOI:** 10.1111/risa.70103

**Published:** 2025-09-10

**Authors:** Benjamin D. Trump, Stephanie Galaitsi, Jeff Cegan, Igor Linkov

**Affiliations:** ^1^ US Army Engineer Research and Development Center Concord Massachusetts USA; ^2^ University of Michigan Ann Arbor Michigan USA; ^3^ Contrcator, US Army Engineer Research and Development Center Concord Massachusetts USA; ^4^ Carnegie Mellon University Pittsburgh Pennsylvania USA

**Keywords:** artificial intelligence | data analytics | emergency response | pandemics | resilience

## Abstract

The COVID‐19 pandemic has exposed critical gaps in our management of systemic risks within complex, interconnected systems. This review examines 10 key areas where artificial intelligence (AI) and data analytics can significantly enhance pandemic preparedness, response, and recovery. Inadequate early warning systems, insufficient real‐time data on resource needs, and the limitations of traditional epidemiological models in capturing complex disease dynamics are among the challenges analyzed. To address these issues, we explore the potential of AI applications, including machine learning‐based surveillance, deep learning for improved epidemiological modeling, and AI‐driven optimization of non‐pharmaceutical interventions. These technologies offer the promise of more timely, accurate, and granular analysis of pandemic risks, thereby supporting evidence‐based decision‐making in rapidly evolving crises. However, implementing AI in pandemic response raises significant ethical and governance challenges, particularly concerning privacy, fairness, and accountability. We parse the promise and challenges of AI in the evolving space of emergency response data analytics and highlight critical steps forward.

## 1 Introduction

The COVID‐19 pandemic has exposed critical gaps in our understanding and management of systemic risks in complex, interconnected systems. From the initial outbreak in Wuhan, China, the SARS‐CoV‐2 virus rapidly spread across the globe, overwhelming healthcare systems, disrupting global supply chains, and triggering widespread socioeconomic upheaval (Nicola et al. 2020). As of June 2024, the pandemic has caused over 10 million deaths worldwide and inflicted trillions of dollars in economic losses. The scale and severity of the crisis have underscored the urgent need for more effective risk analysis and resilience strategies in pandemic emergency management.

A key challenge in the COVID‐19 response was the lack of timely, accurate, and actionable data to inform decision‐making (Ienca and Vayena 2020). Amid rapidly evolving conditions, public health authorities struggled to track the spread of the virus, assess the efficacy of interventions, and allocate scarce resources. Traditional epidemiological models, such as the SEIR (susceptible, exposed, infectious, recovered) model, proved inadequate for capturing the complexity and uncertainty of the pandemic (Holmdahl and Buckee 2020). These models rely on simplifying assumptions and aggregated data that can obscure important heterogeneities and lead to biased predictions (Ioannidis 2020).

Moreover, the COVID‐19 pandemic has highlighted the deeply interconnected nature of systemic risks in the modern world. The virus not only threatened public health but also exposed vulnerabilities in critical infrastructure, such as healthcare facilities, supply chains, and transportation networks (Linkov et al. 2021). The systems of critical infrastructure tend to be highly interdependent, meaning that failures in one domain can cascade across multiple sectors and amplify the overall impact of the crisis (Hynes et al. 2022). For example, shortages of personal protective equipment (PPE) and medical supplies hindered the ability of healthcare workers to treat patients safely, whereas travel restrictions and lockdowns disrupted global trade and exacerbated economic losses (Murano et al. 2021; Ligo et al. 2021).

To address these challenges, there is a growing recognition of the need to integrate artificial intelligence (AI) and data analytics into risk analysis for pandemic emergency management (Bullock et al. 2020). AI encompasses a range of techniques, including machine learning (ML), natural language processing (NLP), computer vision, and deep learning (DL), that can enable more rapid, accurate, and scalable analysis of complex data (Luengo‐Oroz et al. 2020). By leveraging vast troves of structured and unstructured data from multiple sources, such as electronic health records, social media, and environmental sensors, AI can help identify patterns, predict trends, and support decision‐making in real time (Bragazzi et al. 2020). Critically, these tools can help overcome barriers for future pandemics that hindered COVID‐19 response, such as the allocation of scarce resources and healthcare personnel to areas suspected of having a near‐term outbreak or the improved allocation and distribution of government programs to local areas to aid in emergency response and recovery.

Several studies have demonstrated the potential of AI for enhancing various aspects of pandemic response, from forecasting disease spread (Ardabili et al. 2020) to optimizing resource allocation (Lalmuanawma et al. 2020) and developing new treatments and vaccines (Keshavarzi Arshadi et al. 2020). The pandemic has accelerated the adoption of AI and big data analytics in various aspects of public health, from disease surveillance and contact tracing to drug discovery and vaccine development (Alimadadi et al. 2020). Bogoch et al. (2020) used ML algorithms to analyze airline travel data and predict the global spread of COVID‐19 in the early stages of the pandemic. Similarly, Lampos et al. (2021) developed an AI‐powered early warning system that can detect COVID‐19 outbreaks up to a week in advance by monitoring social media posts and online search queries.

However, the application of AI in pandemic risk analysis also raises significant technical, ethical, and governance challenges (Leslie 2020). AI models can be biased, brittle, and difficult to interpret, especially when trained on limited or noisy data (Liang et al. 2022). They can also perpetuate or amplify existing inequities and power imbalances if not designed and deployed with care (Luengo‐Oroz et al. 2021). To realize the full potential of AI in pandemic preparedness and response, we need a more holistic and interdisciplinary approach that considers the social, political, and ethical dimensions of the technology while equally contending with the messy and incomplete data available to analysts at a time when policymakers must act in hours, not days (Palma‐Oliveira et al. 2021).

In this review article, we propose and outline 10 key gaps where AI and data analytics can help improve our systemic understanding and resilience to future pandemics. Based upon observations of COVID‐19 response, these gaps span the entire lifecycle of pandemic emergency management, from early detection and warning to response, recovery, and adaptation. For each gap, we highlight the current challenges and limitations in risk analysis and decision support and discuss how AI and data‐driven approaches can help address these gaps. We argue that by leveraging the power of AI responsibly and in concert with human expertise, we can develop more anticipatory, adaptive, and equitable strategies for managing pandemic risks in an increasingly complex and uncertain world.

## 2 What Did the COVID‐19 Experience Reveal for Emergency Management? Ten Gaps in Data Analytics

The COVID‐19 pandemic has exposed a series of interconnected gaps and challenges in current emergency management practices, particularly when dealing with complex, large‐scale crises. These shortcomings span across multiple domains and highlight the need for a more holistic, adaptive, and equitable approach to risk analysis and resilience. We consider each gap below:

Data acquisition and aggregation gaps

- Public health surveillance and early warning systems
- Real‐time flows of resource needs
- Disaggregated data on socioeconomic impacts for vulnerable populations

Data synthesis gaps

- Characterization of disease parameters
- Models capturing the complexity of disease dynamics
- Systems perspective of risk analysis and resilience planning

Values‐based decision support tools

- Triage protocols
- Optimizing non‐pharmaceutical interventions (NPIs) and early pharmaceutical candidate testing
- Communication and community engagement
- Engaging underserved communities

### 2.1 Data Acquisition and Aggregation Gaps

Data help characterize situations according to understood metrics. When data are unavailable or unreliable, decision‐makers may have trouble making decisions or justifying them. During the COVID‐19 pandemic, serious data gaps emerged that inhibited emergence responses.

Gaps in public health surveillance and early warning systems left healthcare systems unable to plan for future outbreaks. A lack of integration and interoperability of surveillance data across different systems, jurisdictions, and countries hampered efforts to detect and monitor emerging threats (Budd et al. 2020). Many nations, including the United States, lacked the capacity for widespread testing and contact tracing, especially in the early stages of the pandemic, allowing the virus to spread undetected (Hellewell et al. 2020). Perkins et al. (2020) estimated that in the United States, only 1 in 10 to 1 in 20 COVID‐19 cases were being detected in March 2020, due to limited testing and asymptomatic transmission. The reliance on traditional surveillance methods, such as syndromic surveillance and laboratory confirmation, proved insufficient for capturing the full extent of the outbreak in real time (Jombart et al. 2020). The absence of effective early warning mechanisms, such as wastewater surveillance and digital syndromic surveillance, meant that policymakers often had to react to outbreaks after they had already spread widely (Larsen and Wigginton 2020; Sherratt et al. 2021).

As outbreaks unfolded, the lack of real‐time data about resource needs strained healthcare systems worldwide. Hospitals and intensive care units (ICUs) in many countries faced shortages of beds, staff, and equipment (Collier et al. 2021) during surges in COVID‐19 patients. Many healthcare organizations struggled to predict and manage the demand for critical resources accurately due to the lack of granular, real‐time data on patient flows, clinical outcomes, and resource utilization (Klein et al. 2023; O'Reilly‐Shah et al. 2020). The lack of coordination and data sharing among different healthcare providers also hampered efforts to load‐balance and optimize resource allocation across regions (Trentini et al. 2022). For instance, during the first wave of the pandemic in Italy, some regions faced severe shortages of ICU beds, whereas others had spare capacity, but the lack of a centralized system for patient transfers and resource sharing led to suboptimal outcomes (Trentini et al. 2022).

The pandemic revealed insufficient comprehensive and disaggregated data on socioeconomic impacts for vulnerable and marginalized populations, such as racial and ethnic minorities and low‐income communities. Such communities experienced deep inequities in health outcomes and social determinants of health within and across countries, and essential workers bore a disproportionate burden of COVID‐19 infections, hospitalizations, and deaths (Bambra et al. 2020). More data are needed to understand and address such health disparities (Hyder and Razzak 2020). In the United States, the lack of a national standardized system for collecting race and ethnicity data on COVID‐19 cases and deaths made it difficult to track and respond to disparities in real time (Krieger et al. 2020). Many countries lacked the capacity to collect and analyze granular data on key social determinants of health, such as housing conditions, job security, and access to healthcare, which are critical for identifying high‐risk populations and targeting interventions (Berkowitz et al. 2021).

### 2.2 Data Synthesis Gaps

Data alone are insufficient to guide decision‐makers; synthesis is needed to create meaning and relevance within specific decision‐making contexts. A consequence of the data gaps profiled above was an inability to accurately characterize disease parameters, such as the asymptomatic infection rate, the case fatality ratio, and the effectiveness of NPIs like social distancing and mask‐wearing (Saad‐Roy et al. 2020). In the United States, the fragmentation of the public health system, with over 2000 state, local, and tribal health departments using different data standards and reporting mechanisms, made it challenging to aggregate and analyze data at a national level (Campbell et al. 2022).

Given their lack of data and parameters, current epidemiological models were unable to capture the complexity and uncertainty of disease dynamics during the COVID‐19 pandemic. Existing forecasting models, based on historical data and simplistic assumptions about disease progression and patient care pathways, proved inadequate for capturing the nonlinear dynamics and high uncertainty of the pandemic (Ioannidis et al. 2022). Many widely used models, such as the SEIR model, rely on simplifying assumptions about population mixing, transmission rates, and disease progression, which can lead to over‐ or underestimation of key metrics, such as the reproduction number (*R*_0_) and the peak of the epidemic curve (Adam et al. 2020; Holmdahl and Buckee 2020; Rowland et al. 2021). For example, early projections of the COVID‐19 outbreak in the United States, based on the influential Imperial College London model, predicted up to 2.2 million deaths without intervention (Ferguson et al. 2020).

These alarming projections were later revised downward as more data became available and assumptions were refined, but the initial estimates had already shaped public perceptions and policy responses (Engelmann et al. 2023). Furthermore, because data on disease prevalence and transmission dynamics were scarce, researchers faced difficulties calibrating and validating models, leading to wide ranges of uncertainty in projections (Galaitsi et al. 2021). Compounding all this was a lack of standardization and transparency in modeling approaches that hindered comparing and integrating results from different studies and created more obstacles for data‐informed decision‐making (Sridhar and Majumder 2020).

The pandemic highlighted the urgent need to integrate a system perspective into risk analysis and resilience planning, one that accounts for the complex interplay between human health, climate change, biodiversity loss, and other global challenges. The COVID‐19 pandemic also underscored the complex, interconnected nature of systemic risks in the 21st century. The virus not only threatened public health but also exposed the vulnerabilities of critical infrastructure systems, such as healthcare, food supply, and resource production, to cascading failures and disruptions (Hynes et al. 2020). As one example, the COVID‐19 pandemic exposed the fragility and opacity of global supply chains, particularly for critical medical supplies, such as PPE, ventilators, and vaccines.

The sudden surge in demand for these products, coupled with production disruptions and export restrictions, led to widespread shortages, price gouging, and quality control issues (Ivanov 2020). A survey by the US Association for Professionals in Infection Control and Epidemiology found that in March 2020, nearly half of US healthcare facilities were either out of or running low on N95 respirators, and a quarter were facing shortages of face shields (Rebmann et al. 2021). Improving systems understanding can allow decision‐makers to better plan for disruptions and respond proactively as they arise.

### 2.3 Values‐Based Decision Support Tools

When trade‐offs must be made, decision support tools that incorporate societal values can provide timely and actionable information. However, many professionals found themselves facing novel situations with inadequate data or guidance for their responses. We highlight some of the most pressing trade‐offs in this section. The absence of clear, consistent, and ethically sound triage protocols emerged with resource scarcity during surges (Herreros et al. 2020). Many hospitals and health systems had to make difficult decisions about which patients to prioritize for intensive care, often based on ad hoc criteria or subjective judgments (Marincowitz et al. 2024). The ethical debates around triage (Vinay et al. 2021) demonstrate the lack of consensus available for triage decisions. During pandemics, inefficient triage processes can delay or prevent care for the people who most need it (Lai et al. 2020) and use scarce resources inefficiently. Streamlining these processes will save labor and allocate resources more judiciously according to predetermined values.

The challenge of optimizing NPIs to slow the spread of the virus while minimizing social and economic disruption was represented a contentious trade‐off in pandemic management. Many countries struggled to find the right balance between public health benefits and costs when implementing measures such as stay‐at‐home orders, business closures, and travel restrictions (Flaxman et al. 2020). More granular, real‐time data on the effectiveness of different NPIs in different contexts were needed to tailor interventions to local conditions and risk levels (Haug et al. 2020). In turn, the frequent lack of common thresholds to trigger and lift NPIs led to inconsistent and volatile policies across jurisdictions (Hale et al. 2021).

Effective risk communication and community engagement emerged as critical yet challenging aspects of the pandemic response. The rapid spread of misinformation and disinformation through social media and other channels, dubbed an “infodemic” by the World Health Organization (WHO), undermined public trust and compliance with public health measures (Zarocostas 2020). The lack of clear and consistent messaging from public health authorities and political leaders, coupled with the politicization of issues like mask‐wearing and vaccine development, further eroded public confidence (Weingart et al. 2022).

Likewise, the failure to engage and empower underserved and vulnerable communities in the design and implementation of public health interventions led to widening health disparities (Trump et al. 2023). For example, in the United States, Black, Hispanic, and Native American populations experienced disproportionately high rates of COVID‐19 infections, hospitalizations, and deaths, due in part to structural inequities in access to healthcare, housing, and employment (Mackey et al. 2021). The lack of culturally and linguistically appropriate risk communication and community outreach exacerbated these disparities and fueled mistrust in government responses (Farha et al. 2024; Abu Farha et al. 2021). Moreover, the absence of inclusive, participatory governance mechanisms meant that the voices and needs of marginalized communities were often overlooked in the design and implementation of pandemic response policies (Dewidar et al. 2022).

Current risk assessment and management frameworks often treat these issues in isolation, failing to capture their synergistic and compounding effects (IRGC 2018). However, the COVID‐19 pandemic demonstrated their collective importance, and AI‐based innovation provides a viable option for improvement in future pandemic responses. Below we examine how to operationalize these innovations.

## 3 How Can ML, DL, and AI Address Gaps in Current Pandemic Analytics and Response?

Leveraging the power of ML and DL can improve the social, economic, and ethical outcomes of crisis management. The gaps profiled above constitute key limitations of current scientific capability to provide clear, actionable, and defensible guidance for policymakers and the public. Emerging tools, like ML and DL, can help bridge these gaps but equally may pose unique difficulties for future emergency responders. Herein, we consider both the opportunities and potential costs, particularly around issues of privacy, fairness, transparency, and accountability. AI is a broad and multidisciplinary field that develops algorithms and computational systems capable of performing tasks that typically require human intelligence (Wang 2019). These tasks include reasoning, learning, problem‐solving, perception, and language understanding. Within AI, ML and DL are subfields that have shown significant potential in handling complex data‐driven challenges, including those posed by pandemics (Sharma et al. 2021).

ML comprises algorithms that allow computers to learn from and make decisions based on data. Traditional ML methods, such as linear regression, decision trees, and support vector machines (SVMs), rely on structured data and predefined features to make predictions (Maulud and Abdulazeez 2020). These methods have been extensively used in various domains to uncover patterns and insights from data.

DL involves neural networks with many layers (hence “deep”) that can model high‐level abstractions in data (Schmidhuber 2015). DL algorithms, particularly convolutional neural networks (CNNs) and recurrent neural networks (RNNs), have achieved remarkable success in areas such as image and speech recognition, NLP, and bioinformatics. Their ability to learn hierarchical representations from raw data without explicit feature engineering makes them particularly powerful for complex, high‐dimensional data. Below, we provide examples of ML/DL applications to address the gaps profiled above.

### 3.1 Gap: Public Health Surveillance and Early Warning Systems

ML/DL can be applied to the early detection and surveillance of emerging disease outbreaks. Traditional public health surveillance systems, which rely on clinical case reports and laboratory confirmations, often suffer from significant delays and underreporting, especially in resource‐limited settings (Simonsen et al. 2016). ML‐based event‐based surveillance (EBS) methods can complement these systems by mining diverse online data streams (e.g., news articles, social media posts, and search queries) for early signals of disease activity, using NLP and anomaly detection techniques (Jombart et al. 2020). NLP algorithms can be used to monitor social media posts and online search queries for mentions of COVID‐19‐related symptoms, behaviors, and risk perceptions, providing a complementary source of syndromic surveillance data to traditional clinical reporting systems (Yousefinaghani et al. 2021). Similarly, Schwalbe and Wahl (2020) used ML algorithms to analyze satellite imagery and geospatial data to predict the risk of COVID‐19 importation and spread across Africa, based on factors such as population density, mobility patterns, and healthcare infrastructure. BlueDot, a Canadian AI company, used an EBS platform combining NLP, ML, and human expertise to detect the COVID‐19 outbreak in Wuhan, China, on December 31, 2019, several days before the official announcement by the WHO (Niiler 2020). The platform ingested and analyzed large volumes of unstructured data from multiple sources (e.g., news reports, airline ticketing data, and animal disease networks) to identify patterns indicative of an emerging outbreak, which were then validated by epidemiologists. This early warning allowed BlueDot's clients, including government agencies and businesses, to initiate preparedness and response measures ahead of the global spread of the virus.

### 3.2 Gap: Characterizing Disease Parameters in Real Time

Data‐driven AI models, such as DL neural networks and Bayesian hierarchical models, can learn complex nonlinear relationships from large‐scale, high‐dimensional datasets, enabling more accurate and fine‐grained predictions of key epidemiological parameters, such as the time‐varying reproduction number (*R*_t_) and the spatial–temporal spread of the virus (Gnanvi et al. 2021).

The estimation of crucial epidemiological parameters presents a significant challenge in pandemic response due to the dynamic nature of disease transmission and the heterogeneity of populations. Traditional statistical methods often struggle to capture the complex interplay of factors influencing these parameters. However, AI‐driven approaches offer promising solutions to this challenge. ML algorithms, particularly ensemble methods like random forests and gradient boosting machines, can integrate diverse data sources—including demographic information, mobility patterns, and environmental factors—to provide robust estimates of parameters, such as the basic reproduction number (*R*_0_) and the serial interval. These methods can adapt to changing conditions and identify nonobvious correlations that may influence parameter values (Gostic et al. 2020).

DL models, especially RNNs and long short‐term memory (LSTM) networks, excel at capturing temporal dependencies in time‐series data. These models can be employed to estimate time‐varying parameters like *R*_t_, accounting for the impact of interventions and behavioral changes over time. For instance, Xu et al. (2022) developed an LSTM‐based model that outperformed traditional statistical methods in estimating *R*_t_ for COVID‐19 across multiple countries.

Bayesian hierarchical models, when combined with ML techniques, offer a powerful framework for parameter estimation. These models can incorporate prior knowledge, quantify uncertainty, and pool information across different geographical regions or subpopulations. This approach is particularly valuable when data are sparse or of varying quality across different areas, such as with estimating the effectiveness of NPIs on controlling the spread of disease (Flaxman et al. 2020). Furthermore, AI techniques can assist in the calibration and validation of epidemiological models by efficiently exploring high‐dimensional parameter spaces. Techniques, such as Bayesian optimization and active learning, can guide the selection of parameter combinations that best fit observed data, potentially revealing insights into underlying biological and social processes driving disease spread (Dehning et al. 2020).

### 3.3 Gap: Real‐Time Flows of Resource Needs

Throughout the COVID‐19 pandemic, traditional methods of resource management often failed to keep pace with the rapidly evolving demands of the crisis, leading to shortages of essential supplies and suboptimal distribution of healthcare personnel. AI and ML offer powerful tools to address this gap, enabling more dynamic and responsive resource allocation strategies through greater precision of tracking scarce resources throughout their acquisition, use, and disposal.

One key application of AI in this domain is predictive modeling of resource needs. ML algorithms, such as SVMs and random forests, can analyze historical data on hospital admissions, length of stay, and resource utilization to forecast future demands. These models can incorporate a wide range of variables, including local infection rates, demographic data, and even social media trends, to provide more accurate and localized predictions (Mehta et al. 2022). DL approaches, particularly RNNs and transformer models, have also shown promise in capturing complex temporal patterns in healthcare resource utilization. These models can learn from sequences of events and make predictions that account for both short‐term fluctuations and long‐term trends. For example, Borges and Nascimento (2022) developed an LSTM‐based model that accurately predicted ICU bed occupancy up to 10 days in advance, allowing for proactive resource management.

Real‐time optimization of resource allocation is another area where AI can make significant contributions. Reinforcement learning algorithms can be employed to develop adaptive policies for distributing resources across healthcare facilities. These algorithms can learn from ongoing data streams and adjust allocation strategies in response to changing conditions. Komorowski et al. (2018) demonstrated the potential of this approach in optimizing sepsis treatment, and similar principles could be applied to pandemic resource management.

AI can also enhance supply chain management for critical medical resources. NLP techniques can be used to monitor global news sources and social media for early signals of potential supply chain disruptions. ML models can then integrate this information with data on current inventory levels, production capacities, and transportation networks to optimize procurement and distribution strategies (Ivanov and Dolgui 2020). Likewise, computer vision and IoT technologies, combined with AI analytics, can enable real‐time tracking of resource utilization within healthcare facilities. For instance, automated systems can monitor PPE usage, bed occupancy, and equipment availability, providing up‐to‐the‐minute data for resource management decisions. This granular, real‐time data can feed into AI models to continually refine predictions and allocation strategies.

Importantly, AI systems for resource management must be designed with transparency and interpretability in mind. Explainable AI techniques, such as SHAP (SHapley Additive exPlanations) values, can help healthcare administrators to understand the factors driving resource allocation decisions, fostering trust, and enabling human oversight of AI‐driven systems via improving prediction accuracy (Lundberg and Lee 2017).

### 3.4 Gap: Disaggregated Data on Socioeconomic and Health Impacts for Vulnerable Populations

ML models can be used to predict the risk of severe COVID‐19 outcomes, such as ICU admission, mechanical ventilation, and mortality, among hospitalized patients, based on demographic, clinical, and laboratory data (Booth et al. 2021). Nemati et al. (2020) developed an ML model to predict the risk of severe COVID‐19 outcomes (e.g., ICU admission, mechanical ventilation, and mortality) among hospitalized patients, using demographic, clinical, and laboratory data from electronic health records. The model achieved high discrimination (AUC > 0.90) and calibration and identified key risk factors such as age, male sex, and comorbidities (e.g., hypertension, diabetes, and chronic kidney disease). Such prognostic models can help clinicians triage patients, optimize treatment strategies, and allocate scarce resources, such as ICU beds and ventilators, and tailor treatment strategies based on individual prognoses (Nemati et al. 2020).

Beyond clinical outcomes, AI can play a crucial role in understanding and addressing the broader socioeconomic impacts of pandemics on vulnerable populations (Park 2021). NLP techniques can be leveraged to analyze unstructured data sources, such as social media posts, community forums, and news articles, to gain real‐time insights into the challenges faced by vulnerable populations. Sentiment analysis and topic modeling algorithms can be applied to these data streams to track evolving concerns and needs across different communities, enabling more targeted and timely interventions (Gencoglu and Gruber 2020). AI can enhance the integration and analysis of data from multiple sources, including health records, census data, and geospatial information, to create more comprehensive and granular profiles of vulnerability. These integrated datasets can be used to train predictive models that forecast the potential impacts of future pandemics or other crises on specific populations, allowing for more proactive and equitable policy responses, provided that care is taken to avoid and mitigate inequities stemming from the algorithmic interpretation of incomplete evidence regarding underserved and vulnerable populations (Hick et al. 2021).

### 3.5 Gap: Inability of Current Epidemiological Models to Capture the Complexity and Uncertainty of Disease Dynamics

ML/DL techniques can improve the accuracy, flexibility, and interpretability of epidemiological models (Bi et al. 2019). Data‐driven ML models, such as gradient boosting machines, random forests, and SVMs, can learn complex nonlinear relationships between input features (e.g., demographic, environmental, and behavioral factors) and output variables (e.g., disease incidence or reproduction number) directly from large‐scale empirical datasets, without imposing strong parametric assumptions (Bracher et al. 2021). Early efforts at COVID‐19 pandemic modeling caught the attention of the ML epidemiology community. For example, Niazkar and Niazkar (2020) applied artificial neural networks (ANNs) to the task of predicting the COVID‐19 outbreak in multiple countries, including China, Japan, Singapore, Iran, Italy, South Africa, and the United States. Their methodology involved training the ANN models with historical COVID‐19 case data divided into training and testing sets. The study proposed 14 different ANN‐based models, each considering varying time lags of prior daily confirmed cases. They found that models incorporating data from the previous 14 days yielded the most accurate predictions, highlighting the importance of including the virus's incubation period in forecasting models. Similarly, Watson et al. (2021) used a hierarchical Bayesian model with random effects to estimate the time‐varying reproduction number (*R*_t_) of COVID‐19 at the state level in the United States, incorporating data on testing, mobility, and social distancing policies. The model enabled near real‐time monitoring of disease transmission dynamics and the impact of interventions, with uncertainties quantified through posterior credible intervals.

DL techniques can further enhance the predictive power and scalability of epidemiological models by learning spatio‐temporal dependencies and extracting informative features from high‐dimensional, unstructured data sources (e.g., satellite imagery, social media, and electronic health records). Huang et al. (2020) proposed a DL CNN‐based model called DeepFit to forecast the cumulative number of COVID‐19 cases in Chinese provinces, using spatial connectivity patterns and contextual factors as inputs. The model achieved higher accuracy and robustness compared to traditional curve‐fitting methods, especially in scenarios with limited training data. Zeroual et al. (2020) developed a DL model based on LSTM networks to predict the spread of COVID‐19 in six countries, using time‐series data on confirmed cases and fatalities. The model demonstrated strong performance in capturing complex temporal patterns and long‐term dependencies, with the potential to inform resource allocation and policy decisions.

### 3.6 Gap: Systems Perspective of Risk Analysis and Resilience Planning

AI can also play a crucial role in optimizing the allocation and management of scarce healthcare resources during a pandemic. Moreover, AI‐powered supply chain analytics can enable the real‐time monitoring and optimization of medical supply inventories, production capacities, and distribution networks, ensuring that critical resources, such as PPE and vaccines, are delivered to the right place at the right time (Furman et al. 2021; Wells et al. 2021). For instance, IBM's Watson Health unit developed an AI‐based tool that predicts PPE consumption rates and supply chain disruptions for hospitals, based on factors such as local case counts, patient volumes, and inventory levels, allowing healthcare systems to proactively manage their stockpiles and avoid shortages (Snowdon et al. 2021).

AI can enhance systems‐level risk analysis by integrating diverse data streams and modeling complex interdependencies across multiple sectors. ML algorithms, particularly graph neural networks (GNNs), can be employed to model and analyze the intricate relationships between healthcare systems, economic sectors, and social structures. This approach enables a more comprehensive understanding of cascading effects and potential vulnerabilities in the face of pandemic disruptions (Skianis et al. 2023; Panagopoulos et al. 2021). AI can also contribute to the development of adaptive and robust resilience strategies. By leveraging techniques, such as multi‐objective optimization and robust optimization, AI systems can help design resilience plans that balance multiple, often competing objectives (e.g., minimizing health impacts, economic disruption, and social inequalities) while accounting for uncertainties in pandemic progression and intervention effectiveness (Snowdon et al. 2021).

### 3.7 Gap: Absence of Clear, Consistent, and Ethically Sound Triage Protocols

Beyond surveillance and forecasting, ML/DL techniques can also support pandemic response and resource allocation by providing actionable insights from heterogeneous data sources. For example, these risk stratification tools can help clinicians triage patients and prioritize limited resources (Chu et al. 2021).

ML models, trained on large‐scale clinical datasets, can provide rapid, objective assessments of patient risk and potential outcomes, supporting more informed and equitable triage decisions (Ye et al. 2021; Cegan et al. 2021). For instance, Yu et al. (2021) articulate how ML models using XGBoost and CatBoost algorithm sets help hone patient need for mechanical ventilation in COVID‐19 patients with high accuracy, potentially aiding in the allocation of critical care resources as well as minimizing the use of unnecessary and potentially harmful medical interventions to certain patient groups.

However, it is crucial to address potential biases in these AI‐driven triage systems. Techniques from the field of fairness in ML can be employed to audit and mitigate biases related to factors such as race, socioeconomic status, or pre‐existing conditions. For example, Poulain et al. (2023) discuss the use of adversarial debiasing techniques to reduce disparities in the performance of clinical risk prediction models using electronic health records. Explainable AI (XAI) methods, such as SHAP values or LIME (local interpretable model‐agnostic explanations), can enhance the transparency and interpretability of AI‐driven triage decisions. These techniques can provide clinicians with clear insights into the factors influencing the AI's recommendations, facilitating human oversight and allowing for the incorporation of contextual knowledge not captured by the model (Lundberg and Lee 2017).

### 3.8 Gap: Optimizing Non‐Pharmaceutical Interventions, as well as Early Pharmaceutical Intervention Testing

In addition to resource management, ML/DL can support the design and evaluation of non‐pharmaceutical public health interventions, such as social distancing measures, travel restrictions, and vaccination campaigns. Haug et al. (2020) used a hierarchical Bayesian approach to estimate the impact of 6068 NPIs implemented across 79 countries during the early phase of the COVID‐19 pandemic. The model identified several highly effective interventions, such as school closures, gathering bans, and face mask requirements, which reduced the effective reproduction number (*R*_t_) by 10%–20% on average. The study also found that the timing and sequence of interventions played a crucial role in their overall impact, with early and coordinated actions being most effective.

Agent‐based simulation models, powered by ML algorithms, can be used to test the effectiveness of different intervention scenarios in silico before implementing them in the real world. Nguyen et al. (2022) developed a CNN‐based system to automatically detect and track social distancing violations in public spaces, using real‐time video footage from surveillance cameras. The system achieved high accuracy (92%) and real‐time performance (22 fps) in detecting and localizing individuals and calculating their interpersonal distances. These models can incorporate granular data on population demographics, contact networks, and mobility patterns to predict the impact of interventions on disease transmission dynamics and socioeconomic outcomes, allowing policymakers to make more informed and targeted decisions at increasingly granular and local scales (Truszkowska et al. 2021). For example, Kucharski et al. (2020) used an agent‐based model to evaluate the effects of different contact tracing strategies on the control of COVID‐19 in the United Kingdom, finding that manual contact tracing alone would be insufficient to prevent a resurgence of the epidemic, but that a combination of manual and digital tracing, coupled with moderate social distancing measures, could reduce the reproduction number below one. Similarly, Bicher et al. (2022) developed an agent‐based model to optimize COVID‐19 vaccination strategies in Austria, considering factors such as vaccine efficacy, supply constraints, and public acceptance, and found that prioritizing high‐risk groups, such as the elderly and healthcare workers, could significantly reduce mortality and healthcare utilization compared to a random allocation strategy.

Computer vision and DL techniques have also been applied to enforce and monitor compliance with public health measures, such as social distancing and mask‐wearing. Such automated monitoring tools can help public health authorities identify high‐risk locations and target interventions to promote safe behaviors, without requiring manual inspection or physical policing.

ML algorithms have also been used to identify potential drug repurposing candidates for COVID‐19 treatment by learning patterns of drug‐disease associations from large‐scale biomedical knowledge graphs. Zhou et al. (2020) applied a network‐based ML approach to predict 25 promising drug candidates for COVID‐19, including several antivirals (e.g., remdesivir, ribavirin), immunosuppressants (e.g., tocilizumab), and antibiotics (e.g., azithromycin). The predictions were based on the drugs’ network proximity to COVID‐19 target proteins, as well as their co‐expression patterns with other relevant genes. Some of these candidates have since been validated in clinical trials or approved for emergency use, demonstrating the potential of ML‐based drug discovery to accelerate therapeutic development for novel diseases.

### 3.9 Gap: Effective Risk Communication and Community Engagement

Other NLP‐based approaches have been used to monitor the evolving dynamics and impacts of the COVID‐19 infodemic on social media platforms. Cinelli et al. (2020) analyzed over 8 million tweets mentioning COVID‐19 across five languages and found that the volume and sentiment of social media discourse varied significantly across countries and time periods, with misinformation and conspiracy theories often spreading faster and farther than reliable information from official sources. The study highlighted the need for targeted interventions to combat online misinformation and build public trust in science‐based messaging. Chen et al. (2020) developed a multi‐task learning framework based on transformers to classify the information credibility and emotional valence of COVID‐19‐related tweets, achieving high accuracy (>90%) on both tasks.

Another critical challenge exposed by the COVID‐19 pandemic is the spread of misinformation and disinformation on social media platforms, which can undermine public trust in science‐based messaging and hinder compliance with public health measures (Gallotti et al. 2020). AI‐based tools, such as NLP and sentiment analysis, can be used to monitor and counter the spread of false or misleading information online by identifying the sources, narratives, and impact of such content and providing targeted corrections or alternative messaging (Sharma et al. 2020). For instance, the United Nations’ “Verified” initiative used AI algorithms to detect and flag COVID‐19‐related misinformation on social media and then recruited a network of volunteers to create and disseminate fact‐based counternarratives, reaching over one billion people worldwide (Galvão 2021). These AI‐driven tools can help build public trust and resilience to misinformation by ensuring that reliable and authoritative information is easily accessible, engaging, and clearly identified.

## 4 Discussion

The COVID‐19 pandemic has exposed significant gaps and challenges in current approaches to data analytics for pandemic preparedness, response, and recovery. From the limitations of traditional epidemiological models to the lack of real time, granular data on disease spread, resource utilization, and population health impacts, these shortcomings have hindered the ability of decision‐makers to anticipate, detect, and respond effectively to the complex and rapidly evolving threats posed by emerging pathogens. However, the pandemic has also accelerated the development and adoption of AI technologies in public health, offering new opportunities to address these gaps and enhance societal resilience to future outbreaks.

AI can improve the accuracy, timeliness, and granularity of epidemic forecasting models. AI‐driven early warning systems can buy precious time for public health authorities to implement containment and mitigation measures, potentially preventing or delaying the onset of exponential growth in case numbers. By integrating large‐scale, multimodal data sources, AI can identify subtle trends and deviations indicative of an emerging outbreak. These methods enable earlier detection and intervention, reducing event likelihood by allowing targeted responses before widespread transmission occurs. AI‐based models can process mobility patterns, syndromic surveillance, and electronic health records in real time to assess emerging risks dynamically. A key challenge for future ML and DL applications for emergency management includes interpretability, where users would benefit from understanding how their data are used to derive outcomes of AI models and satisfy potential regulatory and legal requirements for equitable use and representation, such as within the United States via the Stafford Act (Vilá et al. 2022). However, the use of AI‐based surveillance and compliance monitoring also raises significant privacy and ethical concerns, which must be carefully addressed through appropriate governance frameworks and public engagement (Ienca and Vayena 2020). For example, the use of facial recognition technologies to identify and track individuals without their consent may violate fundamental rights to privacy and data protection, particularly for marginalized or vulnerable populations (Leslie 2020). The opacity and potential biases of AI algorithms may also lead to unfair or discriminatory outcomes, such as the disproportionate targeting of certain communities for enforcement actions or the insufficient protection of marginalized communities (Satariano and Pifarré Riera 2024). To mitigate these risks, AI‐based pandemic response systems should be designed with transparency, accountability, and fairness in mind, following established principles of ethical AI development and deployment (Floridi et al. 2018).

One promising approach involves affected communities and stakeholders in the design and governance of AI systems through participatory and inclusive processes. For example, the People's Roadmap to a Digital New York City is a community‐driven initiative that seeks to co‐create a vision and principles for the responsible use of digital technologies, including AI, in urban pandemic response (People's Roadmap 2020). The initiative has engaged over 1000 New Yorkers from diverse backgrounds through online surveys, focus groups, and deliberative forums to identify key concerns and recommendations around issues such as data privacy, algorithmic fairness, and digital equity. The resulting roadmap emphasizes the need for community oversight, impact assessments, and public education in the deployment of AI‐based pandemic response tools to ensure that they benefit all New Yorkers and do not exacerbate existing inequalities.

Another key challenge in the ethical development of AI for pandemic response is the lack of diverse and representative datasets, which can lead to biased or skewed models that perform poorly for certain subpopulations. To address this issue, there is a need for concerted efforts to collect and share diverse, high‐quality datasets that capture the full spectrum of COVID‐19 experiences and outcomes, particularly for marginalized and underserved communities. Initiatives such as the National COVID Cohort Collaborative (N3C) aim to create a centralized, harmonized repository of clinical and demographic data from over 1.5 million COVID‐19 patients across the United States, with an emphasis on representativeness and accessibility (Haendel et al. 2021).

In addition to data diversity, the development of AI for pandemic response also requires interdisciplinary collaboration and knowledge integration across domains, such as epidemiology, computer science, social sciences, and ethics. The COVID‐19 pandemic has highlighted the need for cross‐disciplinary teams that can combine technical expertise with contextual understanding to design AI solutions that are not only accurate and efficient but also socially responsible and culturally appropriate (Jit et al. 2021). Likewise, the successful deployment of AI for pandemic response requires robust governance frameworks that can ensure the responsible and accountable use of these technologies, while also fostering innovation and agility in crisis situations. Moreover, reinforcement learning‐based AI models can iteratively refine intervention strategies by continuously learning from epidemiological and policy outcomes. This dynamic capability allows for real‐time adaptation to mitigate vulnerabilities in public health systems. Such frameworks should be based on principles of human rights, transparency, fairness, and privacy protection and should involve multiple stakeholders, including government agencies, civil society organizations, private sector actors, and affected communities (Luengo‐Oroz et al. 2021). For example, the European Commission has proposed a risk‐based regulatory framework for AI that classifies applications into different risk categories, with corresponding requirements for quality, transparency, and oversight (European Commission 2021). The framework also emphasizes the need for human‐centric AI that respects fundamental rights and democratic values and promotes public trust and societal well‐being.

The use of AI in pandemic response also raises important ethical and governance challenges, which must be carefully addressed to ensure that these technologies are developed and deployed in a responsible and equitable manner. For example, the use of AI‐powered surveillance tools, such as contact tracing apps and facial recognition systems, can infringe on individual privacy rights and civil liberties, particularly if they are implemented without adequate transparency, accountability, and oversight (Strelzoff et al. 2024). Moreover, the use of AI algorithms in high‐stakes decisions, such as resource allocation and public health policy, can perpetuate or amplify existing biases and inequities, if they are trained on nonrepresentative or historically biased data (Leslie et al. 2021). Explainable AI (XAI) techniques are essential for ensuring that AI‐generated decisions are interpretable by public health officials, thereby increasing trust in mitigation recommendations and enabling human oversight in critical decision‐making. To mitigate these risks, it is essential to develop and implement ethical frameworks and governance mechanisms that ensure the fair, transparent, and accountable use of AI in pandemic response, based on principles of human rights, nondiscrimination, and public participation (Luengo‐Oroz et al. 2021). This requires the active engagement and collaboration of diverse stakeholders, including public health authorities, civil society organizations, technology companies, and affected communities, to codesign and oversee AI systems that are context‐appropriate, culturally sensitive, and socially beneficial.

Deploying AI in pandemic response requires significant investments in data infrastructure, capacity building, and research and development, particularly in low‐ and middle‐income countries (LMICs) that may lack the resources and expertise to fully harness these technologies (Alami et al. 2020). The COVID‐19 pandemic has highlighted the stark digital divide between high‐ and low‐income settings, with many LMICs facing challenges such as limited access to reliable internet connectivity, electronic health records, and digital surveillance systems. To address these gaps, there is a need for increased international cooperation and support for LMICs to build their data science and AI capabilities through initiatives such as the WHO‐ITU Digital Health Platform (ITU 2022; WHO 2020) or the Africa CDC Institute for Pathogen Genomics (Makoni 2020). These programs aim to provide training, mentorship, and resources to local researchers, engineers, and policymakers, enabling them to develop and apply AI tools that are tailored to their specific contexts and needs.

Ultimately, the COVID‐19 pandemic has underscored the urgent need to leverage AI and data science to enhance pandemic preparedness, response, and recovery. AI‐powered tools and analytics can help address critical gaps in current approaches, such as the lack of granular and timely data, the limitations of traditional epidemiological models, and the challenges of resource allocation and public communication. However, the responsible development and deployment of AI in pandemic response requires a multidisciplinary and inclusive approach that takes into account the complex ethical, social, and political dimensions of these technologies. Key priorities for future pandemic data analysts and policymakers include the following:

1. Investing in the development and validation of AI models that are transparent, explainable, and context‐appropriate, based on diverse and representative datasets.
2. Establishing ethical frameworks and governance mechanisms that ensure the fair, accountable, and participatory use of AI in pandemic response, with a focus on protecting individual rights and promoting health equity.
3. Building data science and AI capacity globally, through international partnerships, training programs, and technology transfer initiatives.
4. Fostering interdisciplinary collaboration and knowledge sharing among experts in public health, data science, social sciences, and bioethics to develop holistic and culturally sensitive AI solutions.
5. Ensure that the data and tools are accessible to a range of stakeholders. Although manipulation of the AI tools and their training sets will be limited to a specific user base, accessing data and outputs is critical to foster trust in government response and recovery for future pandemic efforts.

By learning from the successes and failures of the COVID‐19 response and by harnessing the power of AI in a responsible and inclusive manner, we can build more resilient and equitable health systems that are better prepared to prevent, detect, and respond to future pandemic threats.

## Data Availability

The data that support the findings of this study are available on request from the corresponding author. The data are not publicly available due to privacy or ethical restrictions.

## References

[risa70103-bib-0001] Abu Farha, R. K. , K. H. Alzoubi , O. F. Khabour , and M. A. Alfaqih . 2021. “Exploring Perception and Hesitancy Toward COVID‐19 Vaccine: A Study From Jordan.” Human Vaccines & Immunotherapeutics 17, no. 8: 2415–2420. 34014128 10.1080/21645515.2021.1888633 PMC8475591

[risa70103-bib-0002] Adam, D. C. , P. Wu , J. Y. Wong , et al. 2020. “Clustering and Superspreading Potential of SARS‐CoV‐2 Infections in Hong Kong.” Nature Medicine 26, no. 11: 1714–1719. 10.1038/s41591-020-1092-0 32943787

[risa70103-bib-0003] Alami, H. , L. Rivard , P. Lehoux , et al. 2020. “Artificial Intelligence in Health Care: Laying the Foundation for Responsible, Sustainable, and Inclusive Innovation in Low‐and Middle‐Income Countries.” Globalization and Health 16: 1–6. 32580741 10.1186/s12992-020-00584-1 PMC7315549

[risa70103-bib-0004] Alimadadi, A. , S. Aryal , I. Manandhar , P. B. Munroe , B. Joe , and X. Cheng . 2020. “Artificial Intelligence and Machine Learning to Fight COVID‐19.” Physiological Genomics 52, no. 4: 200–202. 32216577 10.1152/physiolgenomics.00029.2020 PMC7191426

[risa70103-bib-0005] Ardabili, S. F. , A. Mosavi , P. Ghamisi , et al. 2020. “Covid‐19 Outbreak Prediction With Machine Learning.” Algorithms 13, no. 10: 249.

[risa70103-bib-0006] Bambra, C. , R. Riordan , J. Ford , and F. Matthews . 2020. “The COVID‐19 Pandemic and Health Inequalities.” Journal of Epidemiology and Community Health 74, no. 11: 964–968. 32535550 10.1136/jech-2020-214401 PMC7298201

[risa70103-bib-0007] Berkowitz, R. L. , X. Gao , E. K. Michaels , and M. S. Mujahid . 2021. “Structurally Vulnerable Neighbourhood Environments and Racial/Ethnic COVID‐19 Inequities.” Cities & Health 5, no. S1: S59–S62. 35747269 10.1080/23748834.2020.1792069 PMC9216191

[risa70103-bib-0008] Bi, Q. , K. E. Goodman , J. Kaminsky , and J. Lessler . 2019. “What Is Machine Learning? A Primer for the Epidemiologist.” American Journal of Epidemiology 188, no. 12: 2222–2239. 31509183 10.1093/aje/kwz189

[risa70103-bib-0009] Bicher, M. , C. Rippinger , M. Zechmeister , et al. 2022. “An Iterative Algorithm for Optimizing COVID‐19 Vaccination Strategies Considering Unknown Supply.” PLoS ONE 17, no. 5: e0265957. 35499997 10.1371/journal.pone.0265957 PMC9060336

[risa70103-bib-0113] Bogoch, I. I. , A. Watts , A. Thomas‐Bachli , C. Huber , M. U. Kraemer , and K. Khan . 2020. “Potential for Global Spread of a Novel Coronavirus From China.” Journal of Travel Medicine 27, no. 2: taaa011. 31985790 10.1093/jtm/taaa011 PMC7074660

[risa70103-bib-0010] Booth, A. L. , E. Abels , and P. McCaffrey . 2021. “Development of a Prognostic Model for Mortality in COVID‐19 Infection Using Machine Learning.” Modern Pathology 34, no. 3: 522–531. 33067522 10.1038/s41379-020-00700-x PMC7567420

[risa70103-bib-0011] Borges, D. , and M. C. Nascimento . 2022. “COVID‐19 ICU Demand Forecasting: A Two‐Stage Prophet‐LSTM Approach.” Applied Soft Computing 125: 109181. 35755299 10.1016/j.asoc.2022.109181 PMC9212961

[risa70103-bib-0012] Bracher, J. , E. L. Ray , T. Gneiting , and N. G. Reich . 2021. “Evaluating Epidemic Forecasts in an Interval Format.” PLoS Computational Biology 17, no. 2: e1008618. 33577550 10.1371/journal.pcbi.1008618 PMC7880475

[risa70103-bib-0013] Bragazzi, N. L. , M. Mansour , A. Bonsignore , and R. Ciliberti . 2020. “The Role of Hospital and Community Pharmacists in the Management of COVID‐19: Towards an Expanded Definition of the Roles, Responsibilities, and Duties of the Pharmacist.” Pharmacy 8, no. 3: 140. 32784696 10.3390/pharmacy8030140 PMC7558051

[risa70103-bib-0114] Budd, J. , B. S. Miller , E. M. Manning , et al. 2020. “Digital Technologies in the Public‐health Response to COVID‐19.” Nature Medicine 26, no. 8: 1183–1192. 10.1038/s41591-020-1011-4 32770165

[risa70103-bib-0014] Bullock, J. , A. Luccioni , K. H. Pham , C. S. N. Lam , and M. Luengo‐Oroz . 2020. “Mapping the Landscape of Artificial Intelligence Applications Against COVID‐19.” Journal of Artificial Intelligence Research 69: 807–845.

[risa70103-bib-0015] Campbell, T. , A. P. Galvani , G. Friedman , and M. C. Fitzpatrick . 2022. “Exacerbation of COVID‐19 Mortality by the Fragmented United States Healthcare System: A Retrospective Observational Study.” Lancet Regional Health–Americas 12: 100264. 35582265 10.1016/j.lana.2022.100264 PMC9098098

[risa70103-bib-0016] Cegan, J. C. , B. D. Trump , S. M. Cibulsky , et al. 2021. “Can Comorbidity Data Explain Cross‐State and Cross‐National Difference in Covid‐19 Death Rates?.” Risk Management and Healthcare Policy 14: 2877–2885. 34267565 10.2147/RMHP.S313312 PMC8275866

[risa70103-bib-0017] Chen, E. , K. Lerman , and E. Ferrara . 2020. “Tracking Social Media Discourse About the Covid‐19 Pandemic: Development of a Public Coronavirus Twitter Data Set.” JMIR Public Health and Surveillance 6, no. 2: e19273. 32427106 10.2196/19273 PMC7265654

[risa70103-bib-0018] Chu, K. , B. Alharahsheh , N. Garg , and P. Guha . 2021. “Evaluating Risk Stratification Scoring Systems to Predict Mortality in Patients With COVID‐19.” BMJ Health & Care Informatics 28, no. 1: e100389. 10.1136/bmjhci-2021-100389 PMC8441221 34521623

[risa70103-bib-0019] Cinelli, M. , W. Quattrociocchi , A. Galeazzi , et al. 2020. “The COVID‐19 Social Media Infodemic.” Scientific Reports 10, no. 1: 16598. 10.1038/s41598-020-73510-5. 33024152 PMC7538912

[risa70103-bib-0020] Collier, Z. A. , J. M. Keisler , B. D. Trump , J. C. Cegan , S. Wolberg , and I. Linkov . 2021. “Value‐Based Optimization of Healthcare Resource Allocation for COVID‐19 Hot Spots.” In COVID‐19: Systemic Risk and Resilience, 103–114. Springer International Publishing.

[risa70103-bib-0021] Dehning, J. , J. Zierenberg , F. P. Spitzner , et al. 2020. “Inferring Change Points in the Spread of COVID‐19 Reveals the Effectiveness of Interventions.” Science 369, no. 6500: eabb9789. 32414780 10.1126/science.abb9789 PMC7239331

[risa70103-bib-0022] Dewidar, O. , B. A. Kawala , A. Antequera , et al. 2022. “Methodological Guidance for Incorporating Equity When Informing Rapid‐Policy and Guideline Development.” Journal of Clinical Epidemiology 150: 142–153. 35863618 10.1016/j.jclinepi.2022.07.007 PMC9359903

[risa70103-bib-0023] Engelmann, L. , C. M. Montgomery , S. Sturdy , and C. Moreno Lozano . 2023. “Domesticating Models: On the Contingency of Covid‐19 Modelling in UK Media and Policy.” Social Studies of Science 53, no. 1: 121–145. 36227023 10.1177/03063127221126166 PMC9892880

[risa70103-bib-0024] European Commission . 2021. Proposal for a Regulation of the European Parliament and of the Council Laying Down Harmonised Rules on Artificial Intelligence (Artificial Intelligence Act) and Amending Certain Union Legislative Acts. COM(2021) 206 Final . European Commission. https://eur‐lex.europa.eu/legal‐content/EN/TXT/?uri=CELEX%3A52021PC0206.

[risa70103-bib-0025] Farha, F. F. , F. S. Shanto , F. A. Khan , et al. 2024. “Exploring the Changes in Travel Behavior Between the First and Second Waves of the COVID‐19 Pandemic in Dhaka.” Transport Policy 151: 24–35.

[risa70103-bib-0026] Ferguson, N. M. , D. Laydon , G. Nedjati‐Gilani , et al. 2020. Report 9: Impact of Non‐Pharmaceutical Interventions (NPIs) to Reduce COVID‐19 Mortality and Healthcare Demand (Vol. 16) . Imperial College London.

[risa70103-bib-0027] Flaxman, S. , S. Mishra , A. Gandy , et al. 2020. “Estimating the Effects of Non‐Pharmaceutical Interventions on COVID‐19 in Europe.” Nature 584, no. 7820: 257–261. 32512579 10.1038/s41586-020-2405-7

[risa70103-bib-0028] Floridi, L. , J. Cowls , M. Beltrametti , et al. 2018. “AI4People—An Ethical Framework for a Good AI Society: Opportunities, Risks, Principles, and Recommendations.” Minds and Machines 28: 689–707. 30930541 10.1007/s11023-018-9482-5 PMC6404626

[risa70103-bib-0122] Furman, E. , A. Cressman , S. Shin , et al. 2021. “Prediction of Personal Protective Equipment Use in Hospitals During COVID‐19.” Health Care Management Science 24, no. 2: 439–453. 33843005 10.1007/s10729-021-09561-5 PMC8038877

[risa70103-bib-0029] Galaitsi, S. E. , J. C. Cegan , K. Volk , M. Joyner , B. D. Trump , and I. Linkov . 2021. “The Challenges of Data Usage for the United States' COVID‐19 Response.” International Journal of Information Management 59: 102352. 33824545 10.1016/j.ijinfomgt.2021.102352 PMC8017563

[risa70103-bib-0030] Gallotti, R. , F. Valle , N. Castaldo , P. Sacco , and M. De Domenico . 2020. “Assessing the Risks of ‘Infodemics’ in Response to COVID‐19 Epidemics.” Nature Human Behaviour 4, no. 12: 1285–1293. 10.1038/s41562-020-00994-6 33122812

[risa70103-bib-0031] Galvão, J. 2021. “COVID‐19: The Deadly Threat of Misinformation.” The Lancet Infectious Diseases 21, no. 5: e114. 33031753 10.1016/S1473-3099(20)30721-0 PMC7535539

[risa70103-bib-0032] Gencoglu, O. , and M. Gruber . 2020. “Causal Modeling of Twitter Activity During Covid‐19.” Computation 8, no. 4: 85.

[risa70103-bib-0033] Gnanvi, J. E. , K. V. Salako , G. B. Kotanmi , and R. G. Kakaï . 2021. “On the Reliability of Predictions on Covid‐19 Dynamics: A Systematic and Critical Review of Modelling Techniques.” Infectious Disease Modelling 6: 258–272. 33458453 10.1016/j.idm.2020.12.008 PMC7802527

[risa70103-bib-0034] Gostic, K. M. , L. McGough , E. B. Baskerville , et al. 2020. “Practical Considerations for Measuring the Effective Reproductive Number, Rt.” PLOS Computational Biology 16, no. 12: e1008409. 33301457 10.1371/journal.pcbi.1008409 PMC7728287

[risa70103-bib-0035] Haendel, M. A. , C. G. Chute , T. D. Bennett , et al. 2021. “The National COVID Cohort Collaborative (N3C): Rationale, Design, Infrastructure, and Deployment.” Journal of the American Medical Informatics Association 28, no. 3: 427–443. 32805036 10.1093/jamia/ocaa196 PMC7454687

[risa70103-bib-0036] Hale, T. , N. Angrist , A. J. Hale , et al. 2021. “Government Responses and COVID‐19 Deaths: Global Evidence Across Multiple Pandemic Waves.” PLoS ONE 16, no. 7: e0253116. 34242239 10.1371/journal.pone.0253116 PMC8270409

[risa70103-bib-0037] Haug, N. , L. Geyrhofer , A. Londei , et al. 2020. “Ranking the Effectiveness of Worldwide COVID‐19 Government Interventions.” Nature Human Behaviour 4, no. 12: 1303–1312. 10.1038/s41562-020-01009-0 33199859

[risa70103-bib-0038] Hellewell, J. , S. Abbott , A. Gimma , et al. 2020. “Feasibility of Controlling COVID‐19 Outbreaks by Isolation of Cases and Contacts.” Lancet Global Health 8, no. 4: e488–e496. 32119825 10.1016/S2214-109X(20)30074-7 PMC7097845

[risa70103-bib-0039] Herreros, B. , P. Gella , and D. R. de Asua . 2020. “Triage During the COVID‐19 Epidemic in Spain: Better and Worse Ethical Arguments.” Journal of Medical Ethics 46, no. 7: 455–458. 32424063 10.1136/medethics-2020-106352

[risa70103-bib-0120] Hick, J. L. , D. Hanfling , M. K. Wynia , and E. Toner . 2021. “Crisis Standards of Care and COVID‐19: What Did We Learn? How Do We Ensure Equity? What Should We Do?.” NAM Perspectives 2021, 10–31478. 10.31478/202108e PMC8486425 34611605

[risa70103-bib-0040] Holmdahl, I. , and C. Buckee . 2020. “Wrong but Useful—What Covid‐19 Epidemiologic Models Can and Cannot Not Tell Us.” New England Journal of Medicine 383, no. 4: 303–305. 32412711 10.1056/NEJMp2016822

[risa70103-bib-0041] Huang, C. J. , Y. H. Chen , Y. Ma , and P. H. Kuo . 2020. “Multiple‐Input Deep Convolutional Neural Network Model for Covid‐19 Forecasting in China.” MedRxiv 03: 20041608.

[risa70103-bib-0042] Hyder, M. A. , and J. Razzak . 2020. “Telemedicine in the United States: An Introduction for Students and Residents.” Journal of Medical Internet Research 22, no. 11: e20839. 33215999 10.2196/20839 PMC7690251

[risa70103-bib-0116] Hynes, W. , B. Trump , P. Love , and I. Linkov . 2020. “Bouncing Forward: a Resilience Approach to Dealing With COVID‐19 and Future Systemic Shocks.” Environment Systems and Decisions 40, no. 2: 174–184. 32837818 10.1007/s10669-020-09776-x PMC7247742

[risa70103-bib-0043] Hynes, W. , B. D. Trump , A. Kirman , A. Haldane , and I. Linkov . 2022. “Systemic Resilience in Economics.” Nature Physics 18, no. 4: 381–384.

[risa70103-bib-0044] Ienca, M. , and E. Vayena . 2020. “On the Responsible Use of Digital Data to Tackle the COVID‐19 Pandemic.” Nature Medicine 26, no. 4: 463–464. 10.1038/s41591-020-0832-5 PMC7100462 32284619

[risa70103-bib-0045] Ioannidis, J. P. 2020. “Global Perspective of COVID‐19 Epidemiology for a Full‐Cycle Pandemic.” European Journal of Clinical Investigation 50, no. 12: e13423. 33026101 10.1111/eci.13423 PMC7646031

[risa70103-bib-0046] Ioannidis, J. P. , S. Cripps , and M. A. Tanner . 2022. “Forecasting for COVID‐19 Has Failed.” International Journal of Forecasting 38, no. 2: 423–438. 32863495 10.1016/j.ijforecast.2020.08.004 PMC7447267

[risa70103-bib-0047] IRGC . 2018. Guidelines for the Governance of Systemic Risks: In Systems and Organisations in the Context of Transitions. ETH Zurich.

[risa70103-bib-0048] Ivanov, D. 2020. “Predicting the Impacts of Epidemic Outbreaks on Global Supply Chains: A Simulation‐Based Analysis on the Coronavirus Outbreak (COVID‐19/SARS‐CoV‐2) Case.” Transportation Research Part E: Logistics and Transportation Review 136: 101922. 32288597 10.1016/j.tre.2020.101922 PMC7147532

[risa70103-bib-0049] Ivanov, D. , and A. Dolgui . 2020. “Viability of Intertwined Supply Networks: Extending the Supply Chain Resilience Angles Towards Survivability. A Position Paper Motivated by COVID‐19 Outbreak.” International Journal of Production Research 58, no. 10: 2904–2915.

[risa70103-bib-0127] ITU . 2022. Digital Health and Innovation. World Health Organization. https://cdn.who.int/media/docs/default‐source/digital‐health‐documents/who_brochure_dhi_web.pdf?sfvrsn=b37493e2_3&download=true.

[risa70103-bib-0050] Jit, M. , A. Ananthakrishnan , M. McKee , O. J. Wouters , P. Beutels , and Y. Teerawattananon . 2021. “Multi‐Country Collaboration in Responding to Global Infectious Disease Threats: Lessons for Europe From the COVID‐19 Pandemic.” Lancet Regional Health–Europe 9: 100221. 34642675 10.1016/j.lanepe.2021.100221 PMC8495250

[risa70103-bib-0052] Jombart, T. , K. Van Zandvoort , T. W. Russell , et al. 2020. “Inferring the Number of COVID‐19 Cases From Recently Reported Deaths.” Wellcome Open Research 5: 78. 32518842 10.12688/wellcomeopenres.15786.1 PMC7255910

[risa70103-bib-0053] Keshavarzi Arshadi, A. , J. Webb , M. Salem , et al. 2020. “Artificial Intelligence for COVID‐19 Drug Discovery and Vaccine Development.” Frontiers in Artificial Intelligence 3: 65. 33733182 10.3389/frai.2020.00065 PMC7861281

[risa70103-bib-0054] Klein, B. , A. C. Zenteno , D. Joseph , et al. 2023. “Forecasting Hospital‐Level COVID‐19 Admissions Using Real‐Time Mobility Data.” Communications Medicine 3, no. 1: 25. 36788347 10.1038/s43856-023-00253-5 PMC9927044

[risa70103-bib-0119] Komorowski, M. , L. A. Celi , O. Badawi , A. C. Gordon , and A. A. Faisal . 2018. “The Artificial Intelligence Clinician Learns Optimal Treatment Strategies for Sepsis in Intensive Care.” Nature Medicine 24, no. 11: 1716–1720. 10.1038/s41591-018-0213-5 30349085

[risa70103-bib-0055] Krieger, N. , C. Testa , W. P. Hanage , and J. T. Chen . 2020. “US Racial and Ethnic Data for COVID‐19 Cases: Still Missing in Action.” Lancet 396, no. 10261: e81. 33169681 10.1016/S0140-6736(20)32220-0 PMC7581349

[risa70103-bib-0056] Kucharski, A. J. , P. Klepac , A. J. Conlan , et al. 2020. “Effectiveness of Isolation, Testing, Contact Tracing, and Physical Distancing on Reducing Transmission of SARS‐CoV‐2 in Different Settings: A Mathematical Modelling Study.” Lancet Infectious Diseases 20, no. 10: 1151–1160. 32559451 10.1016/S1473-3099(20)30457-6 PMC7511527

[risa70103-bib-0117] Lai, L. , K. A. Wittbold , F. Z. Dadabhoy , et al. (2020. December). “Digital Triage: Novel Strategies for Population Health Management in Response to the COVID‐19 Pandemic.” In Healthcare (Vol. 8, No. 4: p. 100493). Elsevier. 33129176 10.1016/j.hjdsi.2020.100493 PMC7586929

[risa70103-bib-0057] Lalmuanawma, S. , J. Hussain , and L. Chhakchhuak . 2020. “Applications of Machine Learning and Artificial Intelligence for Covid‐19 (SARS‐CoV‐2) Pandemic: A Review.” Chaos, Solitons & Fractals 139: 110059. 32834612 10.1016/j.chaos.2020.110059 PMC7315944

[risa70103-bib-0058] Lampos, V. , M. S. Majumder , E. Yom‐Tov , et al. 2021. “Tracking COVID‐19 Using Online Search.” NPJ Digital Medicine 4, no. 1: 17. 33558607 10.1038/s41746-021-00384-w PMC7870878

[risa70103-bib-0059] Larsen, D. A. , and K. R. Wigginton . 2020. “Tracking COVID‐19 With Wastewater.” Nature Biotechnology 38, no. 10: 1151–1153. 10.1038/s41587-020-0690-1 PMC7505213 32958959

[risa70103-bib-0060] Leslie, D. 2020. “Tackling COVID‐19 Through Responsible AI Innovation: Five Steps in the Right Direction.” Harvard Data Science Review 10: 1–78.

[risa70103-bib-0126] Leslie, D. , A. Mazumder , A. Peppin , M. K. Wolters , and A. Hagerty . 2021. “Does “AI” Stand for Augmenting Inequality in the Era of covid‐19 Healthcare?.” Bmj 372. 10.1136/bmj.n304 PMC7958301 33722847

[risa70103-bib-0061] Liang, W. , G. A. Tadesse , D. Ho , et al. 2022. “Advances, Challenges and Opportunities in Creating Data for Trustworthy AI.” Nature Machine Intelligence 4, no. 8: 669–677.

[risa70103-bib-0062] Ligo, A. K. , E. Mahoney , J. Cegan , et al. 2021. “Relationship Among state Reopening Policies, Health Outcomes and Economic Recovery Through First Wave of the COVID‐19 Pandemic in the US.” PLoS ONE 16, no. 11: e0260015. 34793504 10.1371/journal.pone.0260015 PMC8601438

[risa70103-bib-0063] Linkov, I. , B. D. Trump , M. Golan , and J. M. Keisler . 2021. “Enhancing Resilience in Post‐COVID Societies: By Design or by Intervention?” Environmental Science & Technology 55, no. 8: 4202–4204. 33739817 10.1021/acs.est.1c00444

[risa70103-bib-0064] Luengo‐Oroz, M. , J. Bullock , K. H. Pham , C. S. N. Lam , and A. Luccioni . 2021. “From Artificial Intelligence Bias to Inequality in the Time of COVID‐19.” IEEE Technology and Society Magazine 40, no. 1: 71–79.

[risa70103-bib-0065] Luengo‐Oroz, M. , K. Hoffmann Pham , J. Bullock , et al. 2020. “Artificial Intelligence Cooperation to Support the Global Response to COVID‐19.” Nature Machine Intelligence 2, no. 6: 295–297.

[risa70103-bib-0066] Lundberg, S. M. , and S. I. Lee . 2017. “A Unified Approach to Interpreting Model Predictions,” In Advances in Neural Information Processing Systems. Curran Associates, Inc..

[risa70103-bib-0067] Mackey, K. , C. K. Ayers , K. K. Kondo , et al. 2021. “Racial and Ethnic Disparities in COVID‐19–Related Infections, Hospitalizations, and Deaths: A Systematic Review.” Annals of Internal Medicine 174, no. 3: 362–373. 33253040 10.7326/M20-6306 PMC7772883

[risa70103-bib-0068] Makoni, M. 2020. “Africa's $100‐Million Pathogen Genomics Initiative.” Lancet Microbe 1, no. 8: e318. 35544184 10.1016/S2666-5247(20)30206-8

[risa70103-bib-0069] Marincowitz, C. , T. Stone , P. Bath , et al. 2024. “Accuracy of Telephone Triage for Predicting Adverse Outcomes in Suspected COVID‐19: An Observational Cohort Study.” BMJ Quality & Safety 33, no. 6: 375–385. 10.1136/bmjqs-2021-014382 PMC8983415 35354665

[risa70103-bib-0070] Maulud, D. , and A. M. Abdulazeez . 2020. “A Review on Linear Regression Comprehensive in Machine Learning.” Journal of Applied Science and Technology Trends 1, no. 2: 140–147.

[risa70103-bib-0118] Mehta, N. , and S. Shukla . 2022. “Pandemic Analytics: How Countries Are Leveraging Big Data Analytics and Artificial Intelligence to Fight COVID‐19?.” SN Computer Science 3, no. 1: 54. 34778841 10.1007/s42979-021-00923-y PMC8577168

[risa70103-bib-0071] Murano, Y. , R. Ueno , S. Shi , et al. 2021. “Impact of Domestic Travel Restrictions on Transmission of COVID‐19 Infection Using Public Transportation Network Approach.” Scientific Reports 11, no. 1: 3109. 33542248 10.1038/s41598-021-81806-3 PMC7862278

[risa70103-bib-0072] Nemati, M. , J. Ansary , and N. Nemati . 2020. “Machine‐Learning Approaches in COVID‐19 Survival Analysis and Discharge‐Time Likelihood Prediction Using Clinical Data.” Patterns 1, no. 5: 100074. 32835314 10.1016/j.patter.2020.100074 PMC7334917

[risa70103-bib-0073] Nguyen, T. T. , Q. V. H. Nguyen , D. T. Nguyen , et al. 2022. “Deep Learning for Deepfakes Creation and Detection: A Survey.” Computer Vision and Image Understanding 223: 103525.

[risa70103-bib-0121] Niazkar, H. R. , and M. Niazkar . 2020. “Application of Artificial Neural Networks to Predict the COVID‐19 Outbreak.” Global Health Research and Policy 5, no. 1: 50. 33292780 10.1186/s41256-020-00175-y PMC7680664

[risa70103-bib-0074] Nicola, M. , Z. Alsafi , C. Sohrabi , et al. 2020. “The Socio‐Economic Implications of the Coronavirus Pandemic (COVID‐19): A Review.” International Journal of Surgery 78: 185–193. 32305533 10.1016/j.ijsu.2020.04.018 PMC7162753

[risa70103-bib-0075] Niiler, E. 2020. “An AI Epidemiologist Sent the First Warnings of the Wuhan Virus.” WIRED . February 5. https://www.wired.com/story/ai‐epidemiologist‐wuhan‐public‐health‐warnings/.

[risa70103-bib-0076] O'Reilly‐Shah, V. N. , K. R. Gentry , W. Van Cleve , S. M. Kendale , C. S. Jabaley , and D. R. Long . 2020. “The COVID‐19 Pandemic Highlights Shortcomings in US Health Care Informatics Infrastructure: A Call to Action.” Anesthesia & Analgesia 131, no. 2: 340–344. 32366769 10.1213/ANE.0000000000004945 PMC7219836

[risa70103-bib-0077] Palma‐Oliveira, J. , B. D. Trump , and I. Linkov . 2021. “Why Did Risk Communication Fail for the COVID‐19 Pandemic, and How Can We Do Better?.” In COVID‐19: Systemic Risk and Resilience, 195–211. Springer International Publishing.

[risa70103-bib-0078] Panagopoulos, G. , G. Nikolentzos , and M. Vazirgiannis . 2021. “Transfer Graph Neural Networks for Pandemic Forecasting.” Proceedings of the AAAI Conference on Artificial Intelligence 35, no. 6: 4838–4845.

[risa70103-bib-0079] Park, J. 2021. “Who Is Hardest Hit by a Pandemic? Racial Disparities in COVID‐19 Hardship in the US.” International Journal of Urban Sciences 25, no. 2: 149–177.

[risa70103-bib-0080] People's Roadmap . 2020. The People's Roadmap to a Digital New York City . https://peoples‐roadmap‐nyc.gitbook.io/peoples‐roadmap/. BetaNYC.

[risa70103-bib-0081] Perkins, T. A. , S. M. Cavany , S. M. Moore , R. J. Oidtman , A. Lerch , and M. Poterek . 2020. “Estimating Unobserved SARS‐CoV‐2 Infections in the United States.” Proceedings of the National Academy of Sciences 117, no. 36: 22597–22602. 10.1073/pnas.2005476117 PMC7486725 32826332

[risa70103-bib-0082] Poulain, R. , M. F. Bin Tarek , and R. Beheshti . 2023. “Improving Fairness in Ai Models on Electronic Health Records: The Case for Federated Learning Methods.” In Proceedings of the 2023 ACM Conference on Fairness, Accountability, and Transparency. 1599–1608. ACM. 10.1145/3593013.3594102 PMC10661580 37990734

[risa70103-bib-0083] Rebmann, T. , A. Vassallo , and J. E. Holdsworth . 2021. “Availability of Personal Protective Equipment and Infection Prevention Supplies During the First Month of the COVID‐19 Pandemic: A National Study by the APIC COVID‐19 Task Force.” American Journal of Infection Control 49, no. 4: 434–437. 32858092 10.1016/j.ajic.2020.08.029 PMC7448742

[risa70103-bib-0084] Rowland, M. A. , T. M. Swannack , M. L. Mayo , et al. 2021. “COVID‐19 Infection Data Encode a Dynamic Reproduction Number in Response to Policy Decisions With Secondary Wave Implications.” Scientific Reports 11, no. 1: 10875. 34035322 10.1038/s41598-021-90227-1 PMC8149655

[risa70103-bib-0085] Saad‐Roy, C. M. , C. E. Wagner , R. E. Baker , et al. 2020. “Immune Life History, Vaccination, and the Dynamics of SARS‐CoV‐2 Over the Next 5 Years.” Science 370, no. 6518: 811–818. 32958581 10.1126/science.abd7343 PMC7857410

[risa70103-bib-0086] Satariano, A. , and R. Pifarré Riera . 2024. “An Algorithm Told Police She Was Safe. Then Her Husband Killed Her.” New York Times. https://www.nytimes.com/2024/07/01/world/europe/domestic‐violence‐algorithm.html.

[risa70103-bib-0087] Schmidhuber, J. 2015. “Deep Learning in Neural Networks: An Overview.” Neural Networks 61: 85–117. 25462637 10.1016/j.neunet.2014.09.003

[risa70103-bib-0088] Schwalbe, N. , and B. Wahl . 2020. “Artificial Intelligence and the Future of Global Health.” Lancet 395, no. 10236: 1579–1586. 32416782 10.1016/S0140-6736(20)30226-9 PMC7255280

[risa70103-bib-0090] Sharma, N. , R. Sharma , and N. Jindal . 2021. “Machine Learning and Deep Learning Applications—A Vision.” Global Transitions Proceedings 2, no. 1: 24–28.

[risa70103-bib-0124] Sharma, K. , S. Seo , C. Meng , S. Rambhatla , and Y. Liu . 2020. Covid‐19 on social media: Analyzing misinformation in twitter conversations. arXiv preprint arXiv:2003.12309.

[risa70103-bib-0091] Sherratt, K. , S. Abbott , S. R. Meakin , et al. 2021. “Exploring Surveillance Data Biases When Estimating the Reproduction Number: With Insights Into Subpopulation Transmission of COVID‐19 in England.” Philosophical Transactions of the Royal Society B 376, no. 1829: 20200283. 10.1098/rstb.2020.0283 PMC8165604 34053260

[risa70103-bib-0092] Simonsen, L. , J. R. Gog , D. Olson , and C. Viboud . 2016. “Infectious Disease Surveillance in the Big Data Era: Towards Faster and Locally Relevant Systems.” Journal of Infectious Diseases 214, no. S4: S380–S385. 28830112 10.1093/infdis/jiw376 PMC5144901

[risa70103-bib-0093] Skianis, K. , G. Nikolentzos , B. Gallix , R. Thiebaut , and G. Exarchakis . 2023. “Predicting COVID‐19 Positivity and Hospitalization With Multi‐Scale Graph Neural Networks.” Scientific Reports 13, no. 1: 5235. 37002271 10.1038/s41598-023-31222-6 PMC10066232

[risa70103-bib-0094] Snowdon, J. L. , W. Kassler , H. Karunakaram , B. E. Dixon , and K. Rhee . 2021. “Leveraging Informatics and Technology to Support Public Health Response: Framework and Illustrations Using COVID‐19.” Online Journal of Public Health Informatics 13, no. 1: e1. 33936521 10.5210/ojphi.v13i1.11072 PMC8075350

[risa70103-bib-0095] Sridhar, D. , and M. S. Majumder . 2020. “Modelling the Pandemic.” Bmj 369: m1567. 32317328 10.1136/bmj.m1567

[risa70103-bib-0096] Strelzoff, A. , B. D. Trump , C. L. Cummings , et al. 2024. “Human Intuition and Algorithmic Efficiency Must Be Balanced to Enhance Data Mesh Resilience.” Communications of the ACM 67, no. 5: 48–51.

[risa70103-bib-0115] Trentini, F. , V. Marziano , G. Guzzetta , et al. 2022. “Pressure on the Health‐care System and Intensive Care Utilization During the COVID‐19 Outbreak in the Lombardy Region of Italy: a Retrospective Observational Study in 43,538 Hospitalized Patients.” American Journal of Epidemiology 191, no. 1: 137–146. 34652416 10.1093/aje/kwab252 PMC8549288

[risa70103-bib-0097] Trump, B. D. , A. Jin , S. Galaitsi , et al. 2023. “Equitable Response in Crisis: Methodology and Application for COVID‐19.” ASCE‐ASME Journal of Risk and Uncertainty in Engineering Systems, Part B: Mechanical Engineering 9, no. 3: 031102.

[risa70103-bib-0098] Truszkowska, A. , B. Behring , J. Hasanyan , et al. 2021. “High‐Resolution Agent‐Based Modeling of COVID‐19 Spreading in a Small Town.” Advanced Theory and Simulations 4, no. 3: 2000277. 33786413 10.1002/adts.202000277 PMC7995144

[risa70103-bib-0099] Vilá, O. , G. Smith , B. Cutts , S. Gyawali , and S. Bhattarai . 2022. “Equity in FEMA Hazard Mitigation Assistance Programs: The Role of State Hazard Mitigation Officers.” Environmental Science & Policy 136: 632–641.

[risa70103-bib-0100] Vinay, R. , H. Baumann , and N. Biller‐Andorno . 2021. “Ethics of ICU Triage During COVID‐19.” British Medical Bulletin 138, no. 1: 5–15. 34057458 10.1093/bmb/ldab009 PMC8195142

[risa70103-bib-0101] Wang, P. 2019. “On Defining Artificial Intelligence.” Journal of Artificial General Intelligence 10, no. 2: 1–37.

[risa70103-bib-0102] Watson, G. L. , D. Xiong , L. Zhang , et al. 2021. “Pandemic Velocity: Forecasting COVID‐19 in the US With a Machine Learning & Bayesian Time Series Compartmental Model.” PLOS Computational Biology 17, no. 3: e1008837. 33780443 10.1371/journal.pcbi.1008837 PMC8031749

[risa70103-bib-0103] Weingart, P. , F. van Schalkwyk , and L. Guenther . 2022. “Democratic and Expert Legitimacy: Science, Politics and the Public During the COVID‐19 Pandemic.” Science and Public Policy 49, no. 3: 499–517.

[risa70103-bib-0104] Wells, E. M. , C. L. Cummings , K. Klasa , B. D. Trump , J. C. Cegan , and I. Linkov . 2021. “Real‐Time Anticipatory Response to COVID‐19: A Novel Methodological Approach.” In COVID‐19: Systemic Risk and Resilience, 35–59. Springer International Publishing.

[risa70103-bib-0105] World Health Organization . 2020. Digital Health Platform Handbook: Building a Digital Information Infrastructure (Infostructure) for Health. World Health Organization.

[risa70103-bib-0106] Xu, L. , R. Magar , and A. B. Farimani . 2022. “Forecasting COVID‐19 New Cases Using Deep Learning Methods.” Computers in Biology and Medicine 144: 105342. 35247764 10.1016/j.compbiomed.2022.105342 PMC8864960

[risa70103-bib-0107] Ye, J. , M. Hua , and F. Zhu . 2021. “Machine Learning Algorithms Are Superior to Conventional Regression Models in Predicting Risk Stratification of COVID‐19 Patients.” Risk Management and Healthcare Policy 14: 3159–3166. 34349576 10.2147/RMHP.S318265 PMC8328384

[risa70103-bib-0108] Yousefinaghani, S. , R. Dara , S. Mubareka , A. Papadopoulos , and S. Sharif . 2021. “An Analysis of COVID‐19 Vaccine Sentiments and Opinions on Twitter.” International Journal of Infectious Diseases 108: 256–262. 34052407 10.1016/j.ijid.2021.05.059 PMC8157498

[risa70103-bib-0123] Yu, L. , A. Halalau , B. Dalal , et al. 2021. “Machine Learning Methods to Predict Mechanical Ventilation and Mortality in Patients With COVID‐19.” PLoS ONE 16, no. 4: e0249285. 33793600 10.1371/journal.pone.0249285 PMC8016242

[risa70103-bib-0109] Zarocostas, J. 2020. “How to Fight an Infodemic.” Lancet 395, no. 10225: 676. 32113495 10.1016/S0140-6736(20)30461-X PMC7133615

[risa70103-bib-0110] Zeroual, A. , F. Harrou , A. Dairi , and Y. Sun . 2020. “Deep Learning Methods for Forecasting COVID‐19 Time‐Series Data: A Comparative Study.” Chaos, Solitons & Fractals 140: 110121. 32834633 10.1016/j.chaos.2020.110121 PMC7362800

[risa70103-bib-0111] Zhou, Y. , F. Wang , J. Tang , R. Nussinov , and F. Cheng . 2020. “Artificial Intelligence in COVID‐19 Drug Repurposing.” Lancet Digital Health 2, no. 12: e667–e676. 32984792 10.1016/S2589-7500(20)30192-8 PMC7500917

